# All That Wheezes Is Not Asthma: A Case of Diffuse Large B-Cell Lymphoma of the Larynx

**DOI:** 10.1155/2017/7072615

**Published:** 2017-03-15

**Authors:** Bushra Rahman, Jawad Bilal, Qurat Ul Ain Riaz Sipra, Irbaz Bin Riaz

**Affiliations:** ^1^University of Arizona College of Medicine, Tucson, AZ, USA; ^2^Department of Medicine, University of Arizona, Tucson, AZ, USA; ^3^Department of Hematology & Oncology, University of Arizona, Tucson, AZ, USA

## Abstract

Localized laryngeal lymphoma is a rare entity with an incidence of less than 1% of all laryngeal neoplasms. Diffuse large B-cell lymphoma (DLBCL) is the most common type of laryngeal neoplasms. Here, we describe a case of a young 28-year-old female with large B-cell lymphoma who remained undiagnosed for a long time owing to a myriad of nonspecific presentation including “wheezing.” Although primary laryngeal lymphomas constitute a diagnostic challenge since they are rare, one should have a high index of suspicion for lymphoma of the larynx in patients presenting with unresolved wheezing as it can present catastrophically with acute airway obstruction requiring immediate surgical intervention which was observed in this case. Treatment includes radiotherapy, chemotherapy, immunotherapy, or a combination of these. We hope that the discussions ensuing from case reports regarding uncommon presentations of laryngeal lymphoma may spur the formation of regional/international databases for the description of lymphomas with unusual presentations. This effort can lead to in-depth study of cases and prompt awareness of “rare and subtle presentations” of laryngeal lymphoma.

## 1. Introduction

Localized laryngeal lymphoma is rare with an incidence of less than 1% of all laryngeal neoplasms with diffuse large B-cell lymphoma (DLBCL) being the most common type [1]. Imaging techniques including computed tomography (CT) scan and magnetic resonance imaging (MRI) are helpful diagnostic tools, but the histopathological examination is essential for definite diagnosis. Treatment is nonsurgical with chemotherapy and radiotherapy being the most common modalities. Here, we describe a case of large B-cell lymphoma that remained undiagnosed for a long time owing to a myriad of nonspecific presentations.

## 2. Clinical Presentation

A 28-year-old female with no significant past medical history presented with gradual onset, progressive nonexertional dyspnea, dry cough, and wheezing for 4 months. Review of symptoms was positive for low-grade fever, chills, night sweats, lightheadedness, and dizziness. She denied chest pain, hemoptysis, weight loss, sick contacts, leg swelling, or recent travel. She was treated with multiple courses of systemic steroids without much relief under the diagnostic impression of bronchial asthma exacerbations. Home medications included as needed albuterol and prednisone. Her family and social history were noncontributory. On physical examination, temperature was 36.3°C, blood pressure 126/82 mmHg, pulse 118/min, and respirations 21/min. Lungs examination revealed bilateral expiratory and inspiratory wheezing with increased work of breathing.

Complete blood count was significant for leukocytosis (16.9 × 10^9^/L) and normocytic anemia (hemoglobin 11.7 g/dL). The comprehensive metabolic panel was unremarkable. Chest X-ray was normal. Serum beta 2 microglobulin and LDH levels were 1.60 (mg/L) and 119 (U/L), respectively. The computed tomography angiography (CTA) done outside hospital demonstrated large mass involving the upper esophagus, larynx, or thyroid gland encasing the upper airway causing the mass effect and narrowing on the upper trachea. It was deviating the trachea anteriorly and to the right. There was no evidence of pulmonary emboli. MRI soft tissue neck done at our facility showed mass lesion of posterior hypopharyngeal wall and subglottic region that extends to the medial portion of thyroid gland bilaterally. Computed tomography (CT) abdomen/pelvis with contrast revealed ongoing bibasilar atelectasis with nonspecific bronchial wall thickening.

ENT service was consulted. Emergent tracheostomy and direct laryngoscopy with subglottic biopsy were performed which significantly improved her symptoms. The direct laryngoscopy identified a mucosa covered soft tissue mass in the subglottic region of posterior upper trachea that was approximately 3 cm in size. Gross morphology of subglottic biopsy demonstrated intermediate to large cells with irregular nuclear contours, dispersed chromatin, and occasional mitotic figures. Immunohistochemistry demonstrated large lymphocytes representing CD20 positive B-cells which were partially positive for PAX5, CD45, and Bcl-6 and negative for CD3, CD5, CD10, CD15, CD 30, CD 34, CD 43, and Bcl-2. No monoclonal B-cells were detected by flow cytometry of bone marrow biopsy specimen and cytogenetic analysis demonstrated no evidence of acquired clonal abnormality. The positron emission tomography (PET) from vertex of skull to thighs and computed tomography (CT) of head, neck, chest, and abdomen/pelvis without contrast were only significant for a hypermetabolic retropharyngeal soft tissue mass measuring 3 cm × 1 cm anteroposteriorly.

The patient was diagnosed with high-grade diffuse large B-cell lymphoma germinal center B-cell (GCB) subtype. The clinical stage was 1E per Ann Arbor staging system. The IPI score was 0. Oncology team was consulted. She was started on dexamethasone and discharged to outpatient oncology clinic for R-CHOP (rituximab, cyclophosphamide, doxorubicin, vincristine, and prednisone) chemotherapeutic regimen. Since discharge, she has received the three cycles of R-CHOP therapy without complications. Repeat PET/CT scan was done after completion of 3 cycles that showed complete resolution of hypermetabolic activity in neck and no other significant abnormalities were reported. She was referred to radiation oncologist for radiotherapy but patient did not follow up and refused further treatment. She was encouraged to receive the radiation therapy and continue to follow up in oncology clinic regularly.

## 3. Discussion

Primary non-Hodgkin lymphoma (NHL) of the larynx was first reported by MacKenty in 1934 [2]. The mean age of occurrence is seventh decade with male predominance [3]. Commonly reported symptoms include dyspnea, dysphagia, dysphonia, hoarseness, and cervical lymphadenopathy. A significant diagnostic delay is commonly observed secondary to subtle and nonspecific presentation. But, rarely it may present catastrophically with acute airway obstruction requiring immediate surgical intervention which was observed in this case.

NHL is commonly localized in the supraglottic area and subglottic areas where lymphoid tissue predominates. It can also involve surrounding structures including salivary glands, thyroid, nasopharynx, and tonsils. Dissemination may eventually occur to other sites in the respiratory tract, stomach, lung, orbit, or skin after a long disease-free interval [4]. The diffuse large B-cell and the mucosa-associated lymphoid tissue- (MALT-) type marginal zone B-cell lymphomas are the most commonly found primary laryngeal hematopoietic neoplasms [5]. A comprehensive literature search revealed fewer than 100 cases to date out of which 35 cases are true stage IV tumors of the larynx.

Staging of non-Hodgkin B-cell lymphoma is done using the Ann Arbor staging system and includes stages I–IV, presence or absence of systemic symptoms, and the involvement of extranodal sites. Our patient was staged as 1E as she only had a single isolated site of extranodal disease. A surgical excision biopsy remains the optimal method of diagnosis and allows assessment of nodal architecture and provides adequate material for phenotypic and molecular studies.

Lugano classification confirms the use of fluorodeoxyglucose positron emission tomography (FDG-PET)/CT scan as the gold standard for staging diffuse large B-cell lymphoma (DLBCL) [6]. PET/CT is more accurate than contrast-enhanced CT (CeCT), with increased sensitivity for nodal and extranodal sites, although CeCT is often carried out before PET/CT in clinical practice. Focal bone marrow FDG uptake with or without increased diffuse uptake is more sensitive than bone marrow biopsy (BMB) for infiltration in DLBCL and is highly specific [7]. The International Prognostic Index (IPI) has been the primary prognostic model used in the management of patients with DLBCL since its publication in 1993 [8]. The addition of rituximab to CHOP chemotherapy has resulted in a marked improvement in outcome for patients with DLBCL and has altered what was previously understood regarding risk assessment. Currently, the revised International Prognostic Index (R-IPI) is a better predictor of outcome than the standard IPI for patients with diffuse large B-cell lymphoma treated with R-CHOP and allows for better treatment planning [9].

Treatment for NHL includes radiotherapy, chemotherapy, immunotherapy, or a combination of these. Coiffier and his colleagues demonstrated that immunotherapy in the form of rituximab along with CHOP regimen has a significant role in the treatment of this tumor leading to increased rates of complete response, decreased rates of treatment failure and relapse, and improved overall survival as opposed to CHOP alone [10]. Coiffier et al. in a recent study of 399 patients, aged 60–80 years, have also shown that survival end points were improved in patients treated with R-CHOP: the 10-year progression-free survival was 36.5%, compared with 20% with CHOP alone, and the 10-year overall survival was 43.5% compared with 27.6%. Relapses occurring after 5 years represented 7% of all disease progressions in the study. Thus, the results from the 10-year analysis confirmed the benefits and tolerability of the addition of rituximab to CHOP [11].

However, approximately 40% of patients that have been treated with R-CHOP will have refractory disease or disease that will relapse after an initial response, and the majority of patients with relapsed DLBCL will succumb to the disease [10, 12]. Gene expression profıling (GEP) of DLBCL has resulted in the identifıcation of two major and clinically distinct subtypes that are classifıed based on cell of origin (COO) and are associated with differences in clinical outcome: germinal center B-cell (GCB) and non- GCB (includes ABC (activated B-cell) and primary mediastinal B-cell types). The GCB subset of DLBCL is associated with better outcomes and may require different therapeutic approaches whereas ABC DLBCL subtype is associated with poor outcome. The 5-year PFS rate is reported at 40% in ABC DLBCL and 74% in GCB DLBCL with R-CHOP [13]. Additionally, double-hit lymphomas (approximately 5% to 10% of patients) and double-expressor lymphomas, which overexpress MYC and BCL2 protein, are aggressive DLBCLs and are also associated with a poor prognosis [14]. Since our patient was identified to have GCB subtype of DLBCL, she is expected to have better outcomes as compared to patients with non-GCB subtype of DLBCL.

Based on current available data, indications for radiotherapy include bulky disease (>7.5 cm), skeletal involvement, and partial response to immunochemotherapy among patients with nonbulky disease [15]. In a recent study by Held et al., radiotherapy was used to debulk extralymphatic disease which showed improved outcomes of elderly patients with aggressive B-cell lymphoma [16].

Diffuse large B-cell Lymphoma is heterogenous clinically, morphologically, and molecularly and hence treatment is variable depending upon staging and extent of involvement. Based on the SWOG studies, it is recommended to use 3 cycles of R-CHOP with involved field radiation, for most patients with stage I and nonbulky stage II disease [17]. Our patient also received 3 cycles of R-CHOP and was advised radiation therapy as follow-up as she was staged as 1E per the Ann Harbor system. Six cycles of R-CHOP chemotherapy without radiation appear to be another effective option in patients without bulk disease, based upon the MInT study results [18]. Patients with bulky disease clearly require more chemotherapy and possibly benefit from intensified regimens, as suggested by the GELA study [19]. As more insight is gained from Gene Expression Profiling, it is anticipated that novel therapeutic approaches will be developed for the high risk subset of patients.

Overall five-year survival of patients with NHL is 43 percent for low-grade and 48 percent for high-grade lesions [20]. For extranodal disease, five-year survival is 54 per cent and that for nodular disease is 65 per cent [21]. Follow-up strategy in patients with NHL includes a recommendation for careful history and physical examination every 3 months for 1 year, every 6 months for 2 more years, and then once a year with attention to development of secondary tumors or other long-term side-effects of chemotherapy. Blood count should be carried out at 3, 6, 12, and 24 months, then only as needed for evaluation of suspicious symptoms or clinical findings in those patients suitable for further therapy. Minimal radiological examinations at 6, 12, and 24 months after end of treatment by CT scan are common practice, but there is no definitive evidence that routine imaging in patients in complete remission provides any outcome advantage, and it may increase the incidence of secondary malignancies [22]. Routine surveillance with PET scan is not recommended [23].

## 4. Conclusion

The primary laryngeal lymphomas may present as a diagnostic challenge since they are rare and can present with nonspecific respiratory symptoms. The information ensuing from this case and others can help in the formation of regional/international database regarding unusual presentations of laryngeal lymphomas. Meanwhile, it is imperative that physicians should be cognizant of such rare presentations and be vigilant to evoke the diagnosis systematically considering potentially fatal outcomes.
